# Quantitative assay for farnesol and the aromatic fusel alcohols from the fungus *Candida albicans*

**DOI:** 10.1007/s00253-022-12165-w

**Published:** 2022-09-15

**Authors:** Cory H. T. Boone, Daniel J. Gutzmann, Jaxon J. Kramer, Audrey L. Atkin, Kenneth W. Nickerson

**Affiliations:** School of Biological Sciences, University of Nebraska, Lincoln, NE USA

**Keywords:** Farnesol, Aromatic fusel alcohols, *Candida albicans*, Quorum sensing, Fungal signaling

## Abstract

**Abstract:**

The dimorphic fungus *Candida albicans* is a commensal and opportunistic fungal pathogen of humans. It secretes at least four small lipophilic molecules, farnesol and three aromatic fusel alcohols. Farnesol has been identified as both a quorum sensing molecule (QSM) and a virulence factor. Our gas chromatography (GC)-based assay for these molecules exhibits high throughput, prevention of analyte loss by avoiding filtration and rotary evaporation, simultaneous cell lysis and analyte extraction by ethyl acetate, and the ability to compare whole cultures with their cell pellets and supernatants. Farnesol synthesis and secretion were separable phenomena and pellet:supernatant ratios for farnesol were high, up to 12:1. The assay was validated in terms of precision, specificity, ruggedness, accuracy, solution stability, detection limits (DL), quantitation limits (QL), and dynamic range. The DL for farnesol was 0.02 ng/µl (0.09 µM). Measurement quality was assessed by the relative error of the whole culture versus the sum of pellet and supernatant fractions (WPS). *C. albicans* strain SC5314 grown at 30 °C in complex and defined media (YPD and mRPMI) was assayed in biological triplicate 17 times over 3 days. Farnesol and the three aromatic fusel alcohols can be measured in the same assay. The levels of all four are greatly altered by the growth medium chosen. Significantly, the three fusel alcohols are synthesized during stationary phase, not during growth. They are secreted quickly without being retained in the cell pellet and may accumulate up to mM concentrations.

**Key points:**

• *Quantitative analysis of both intra- and extracellular farnesol, and aromatic fusel oils*.

• *High throughput, whole culture assay with simultaneous lysis and extraction*.

• *Farnesol secretion and synthesis are distinct and separate events*.

**Supplementary Information:**

The online version contains supplementary material available at 10.1007/s00253-022-12165-w.

## Introduction

The dimorphic fungus *Candida albicans* is a major commensal and opportunistic fungal pathogen of humans (Rai et al. 2021). *E,E*-Farnesol, further referred to simply as farnesol, is a signaling molecule secreted by aerobic *C. albicans*. It was first discovered as a quorum sensing molecule (QSM) for *C. albicans* where it acted by blocking germ tube formation (GTF) and the conversion of yeasts to mycelia (Hornby et al. 2001). Since then, it has been shown to act as an antifungal agent versus other potentially competing fungi (Machida et al. 1998; Semighini et al. 2006), as well as a virulence factor for pathogenicity in mice (Navarathna et al. 2007). Farnesol acts as a chemoattractant for both macrophages (Hargarten et al. 2015) and neutrophils (Zawrotniak et al. 2019), with the farnesol being detected by the TLR2 receptors of the two cell types (Ghosh et al. 2010; Zawrotniak et al. 2019). Examination of eight different *Candida* species found that only *C. albicans* and *C. dubliniensis* secreted farnesol (Weber et al. 2008). Critically, farnesol blocks biofilm formation in both *C. albicans* (Ramage et al. 2002) and *C. dubliniensis* (Jabra-Rizk et al. 2006). We concluded that farnesol secretion is a nearly universal feature of clinical isolates of *C. albicans* (Hornby and Nickerson 2004). The seven strains of *C. albicans* studied by Weber et al. (2008) varied fourfold in terms of the levels of farnesol secreted. Later, Jung et al. (2016) compared 149 isolates of *C. albicans* from blood stream infections in ten Korean hospitals; they found that all of them produced farnesol and that their levels of maximum production varied by 70-fold. There have been numerous papers showing positive and negative interactions between farnesol and a wide variety of antibiotics used to treat patients with candidiasis. In sum, by 2016, there were ca. 250 papers and 22 patents dealing with *C. albicans* and farnesol (Nickerson and Atkin 2017). Despite this level of interest over the past 21 years, there are many basic features about farnesol biology and signaling which are still poorly understood (Polke et al. 2018). These include such major questions as the synthesis, regulation, and mode of action of farnesol, as well as how it is transported in and out of the cell.

Effective approaches to answer these questions require reliable, accurate, and cost-effective assays for farnesol measurement. The need for this straightforward goal is reinforced by the large variability in farnesol measurements reported by different laboratories. For instance, our lab typically reported 2–4 µM farnesol while Weber et al. (2008; 2010) reported 40–50 µM. Also, the farnesol levels detected by different individuals in our lab often varied greatly from year to year. We recently traced these variances to whether the culture supernatants had been filter-sterilized, and the chemical nature of the filters employed. Given the magnitude of the differences observed, we thought it necessary to design an entirely new assay for farnesol. Of critical importance for our method development was our discovery that the stickiness of farnesol meant that all filtration steps should be avoided while the volatility of farnesol meant that all chances for evaporation should be minimized. Our new method provides an accurate and reliable, validated tool that assays and compares whole cultures of *C. albicans* with their cell bound (pellet) and secreted (supernatant) fractions. In doing so, we have a platform with the capacity to answer the above-mentioned outstanding questions, in addition to fostering new ideas. Using this assay we have developed several ideas on the induction of hyphae/germ tubes in *C. albicans*, and the relationships between farnesol and the aromatic fusel alcohols, as well as showing that the synthesis and secretion of farnesol are distinct and separable phenomena.

## Materials and methods

### Organism, growth media, and growth conditions

The strain used throughout method development and validation was C. *albicans* SC5314 (Hickman et al. 2013). Three growth media were employed. Early experiments were conducted in GPP, a minimal defined glucose-phosphate-proline medium (Kulkarni and Nickerson 1981). Most of our work compared the farnesol and fusel alcohols from cells grown in either YPD or RPMI-1640, two media commonly used for the growth of *C. albicans*. YPD is a complex medium containing 1% yeast extract, 2% peptone, and 2% dextrose while RPMI-1640 is a defined medium containing most amino acids and vitamins. It was originally formulated to support growth of cells in tissue culture and thus was intended for use with a serum supplement. We used R7755 (Millipore-Sigma, St. Louis, MO, USA) to which we added the recommended levels of glucose, L-glutamine, and MOPS (3-(*N*-morpholino)propanesulfonic acid) (Gulati et al. 2018). Significantly, we also added 250 µL/L of the mineral mix for GPP (Kulkarni and Nickerson 1981), providing a final concentration of ca. 1 mg/L of Cu(II), Zn(II), Mn(II), and Fe(II). Addition of the mineral mix was essential. In a direct comparison of growth in RPMI-1640 with varying amounts of glutamine or ammonium tartrate, after 16 h at 30 °C the 10 flasks without added minerals achieved an average optical density of OD_600_ = 0.90 whereas the four flasks with added mineral mix achieved an average optical density of OD_600_ = 7.1. In this paper, the modified RPMI-1640 with added minerals (mRPMI) was used for all the cell growth studies while unmodified RPMI was used for validation studies not requiring cell growth.

Growth curves were performed in Fernbach flasks containing 1 L of growth medium aerated by shaking at 225 rpm (rounds per minute) at 30 °C. Following inoculation with overnight cultures to an initial OD_600_ of 0.05 for YPD and 0.25 for mRPMI, log phase started at 6 h whereupon two 10 mL samples were harvested at 90 min intervals from T_6_ to T_24_ and at 12-h intervals from T_24_ to T_72_. Analyte measurements were made on the whole culture (cell-free and cell-associated), cell pellet (cell-associated), and supernatant (cell-free). Analyte concentration was normalized to cell density via their OD_600_ values (SpectraMax 384 Plus, Molecular Devices, San Jose, CA, USA).

### Chromatographic conditions and open lab data analysis

Experiments were performed on Agilent Technologies 7890 B gas chromatography system (Santa Clara, CA, USA) equipped with autosampler and flame ionization detector (FID). Chromatographic separation was performed on an HP-Innowax (Agilent 19091 N-133I) column with a film thickness of 0.25 µm, diameter of 0.25 mm, and length of 30 m. An injection volume of 2 µL was used (splitless) with hydrogen as carrier gas at an initial temperature of 90 °C ramped at a rate of 30 °C/min to a final temperature of 245 °C and held there for 7 min. Data files were batched and analyzed using OpenLab Services (Agilent Technologies) with software calculated response factors based on standard calibrator solutions. Quantitation settings were calculated using the peak area from the 17.2 ng/µL 1-tetradecanol internal standard.

### Preparation of standard solutions

A 4 mM 1-tetradecanol internal standard stock solution was prepared by dissolving 430 mg of 1-tetradecanol in 500 mL ethyl acetate. Two 100 × analyte standard solutions, a low concentration standard (standard A) and a high concentration standard (standard B) in methanol were prepared fresh weekly throughout this study. Standards A and B contained 4000 and 10,000 ng/µL methionol (3-(methylthio)-1-propanol, CAS: 505–10-2, Millipore-Sigma), 200 and 4000 ng/µL phenethyl alcohol (2-phenylethanol, CAS: 60–12-8, Millipore-Sigma), 100 and 500 ng/µL farnesol (trans,trans-farnesol 96%, CAS: 106–28-5, Millipore-Sigma), 1500 and 4500 ng/µL tyrosol (2-(4-hydroxyphenyl)ethanol, CAS: 501–94-0, Millipore-Sigma), and 500 and 2000 ng/µL tryptophol (3-(2-hydroxyethyl)indole, CAS: 526–55-6, Millipore-Sigma), respectively. Both standards were prepared using fresh 5000 × methionol, 10,000 × phenethyl alcohol, 1000 × tyrosol and tryptophol working stocks, and fresh 10,000 × (45 mM) farnesol. Internal standard stock solution was stable for at least 6 months and this stability was confirmed for each new extraction solution batch via extraction of blank media sample. All stock solutions were stored at − 20 °C.

### Fractionation and measurement quality assessment

**Fig. 1:**
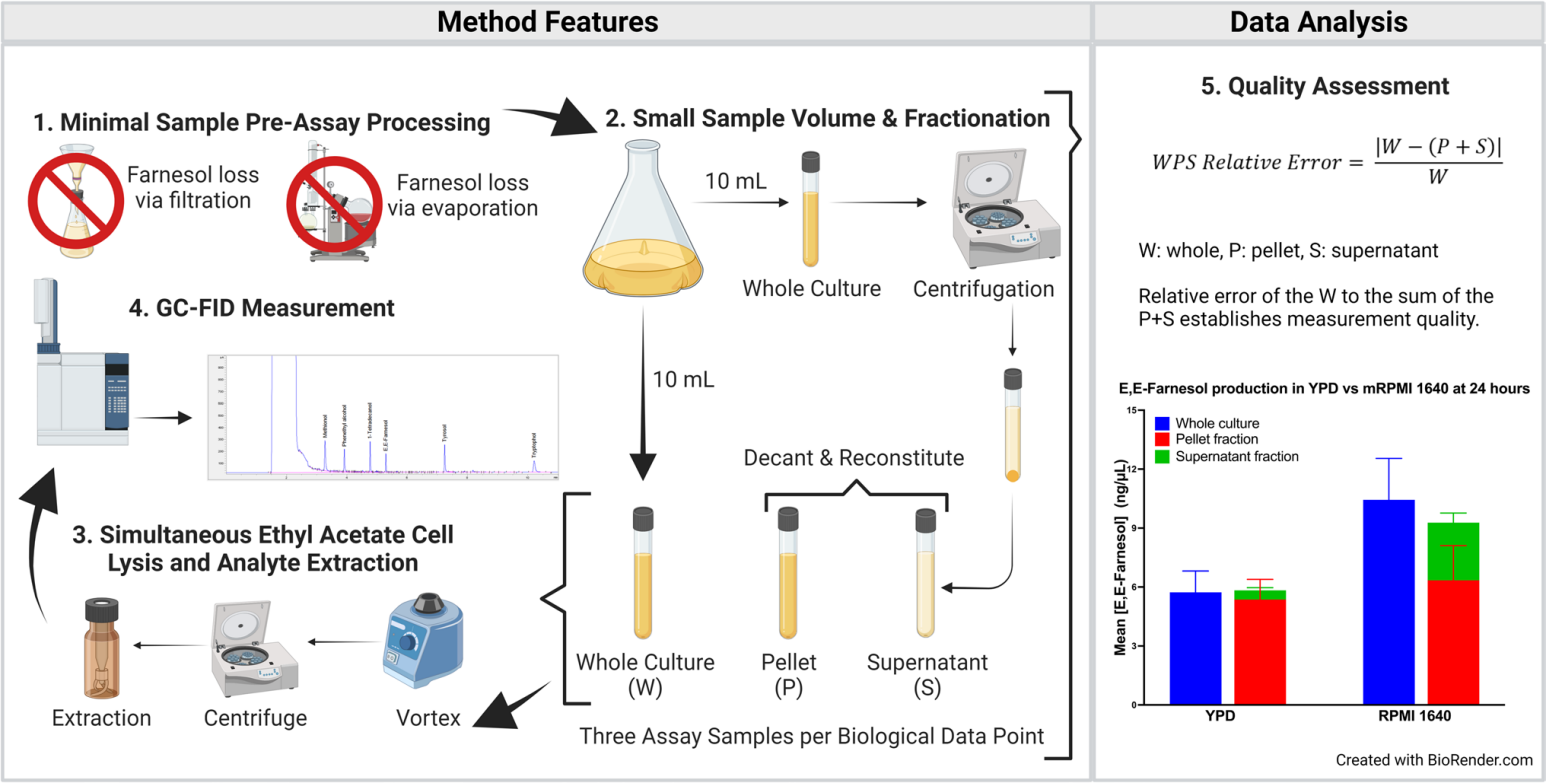
Workflow for quantitative analysis of farnesol and the aromatic fusel alcohols. Key steps include: (1) avoiding filtration and evaporation, (2) sample harvest and fractionation, (3) simultaneous cell lysis and extraction with ethyl acetate, (4) GC-FID sample measurement, (5) data measurement quality assessment by comparison of the whole culture values (W) with those for cell pellets (P) and supernatants (S)

Two ten mL aliquots were harvested from culture into glass screw cap test tubes (15 × 123 mm). One aliquot was directly extracted and measured for analyte concentrations; this we refer to as the whole culture (W). The second aliquot was centrifuged for 5 min at 2000 rpm in a JS7.5 swinging bucket rotor in a Beckman model J2-21 centrifuge (Beckman Coulter Life Sciences, Indianapolis, IN, USA). After centrifugation, the cell-free fraction was transferred into a clean tube; this we refer to as the supernatant fraction (S). The pelleted cells were reconstituted in 10 mL fresh medium by vortexing for 2 min. This sample is the pellet fraction (P). It is important that the pellet not be too tightly packed (only 2000 rpm for 5 min) so that the pelleted cells can be fully resuspended. The pellet and supernatant fractions were subsequently extracted and measured for their analyte concentrations. The relative error for these three values reflected the quality of fractionation and analyte measurement for each biological data point, using the following Eq. 1:

1 $$\mathrm{WPS}\;\mathrm{Relative}\;\mathrm{Error}=\frac{\left|\mathrm W-(\mathrm P+\mathrm S)\right|}{\mathrm W}$$

where *W* = whole culture in ng/µL, *P* = pellet fraction in ng/µL, and *S* = supernatant fraction in ng/µL. We treat *W* as the expected value and *P* + *S* as the observed value. The values are absolute because *W* − (*P* + *S*) can be negative. Figure 1 shows a single time point (24 h) for YPD and mRPMI grown cells, comparing the farnesol content of a whole culture versus the sum of the supernatant and pellet fractions. They are close to identical, meaning a whole-pellet-supernatant (WPS) relative error value from Eq. 1 of close to zero.

### Extraction and cell lysis

Extraction solution was prepared by adding 2 mL of 4 mM 1-tetradecanol stock solution to 98 mL ethyl acetate for a 1-tetradecanol assay concentration of 17.2 ng/µL. The extraction solution (1.5 mL) is added to 10 mL of sample in glass screw cap tubes. Each tube is covered with Duraseal® (Diversified Biotech, Boston, MA, USA), capped, and vortexed on high for 2 min inverting intermittently. Then 1.0 mL of 5 M NaCl is added per tube followed by vortexing for an additional 10 s and the extraction tubes are centrifuged at 3000 rpm for 12 min in a Beckman J2-21 centrifuge with a JS 7.5 swinging bucket rotor. The organic phase is removed with individual glass Pasteur pipets into 2 mL amber autosampler vials (Agilent Technologies 5182–0716) with PTFE/red silicone septa screw caps (Agilent Technologies 5185–5820) and 50 µl inserts (JG Finneran Associates, Vineland, NJ, USA, 4005BS-625) and stored for up to one week at − 20 °C until assayed by gas chromatography. An extraction ratio of 1.5 mL per 10 mL sample was used for all experiments in this paper in order to achieve optimal sensitivity. With the use of standard calibrator solutions and internal standard, assay volumes could be adjusted for experimental optimization.

### Loss of farnesol by evaporation

Fifty mL of fresh GPP media in 250 mL flasks was spiked with 2 × standard A and treated as an inoculated culture by shaking at 225 rpm at 30 °C. The flasks were either covered with a foam plug or sealed with Duraseal® and Parafilm® (Bemis Flexible Packaging, Neenah, WI, USA). An aliquot of media was taken prior to shaking to establish a baseline and then the cell-free cultures were assayed at 24-, 48-, and 72-h time points. Percent reduction values are presented as the mean of four independent experiments ± standard deviation.

### Loss of farnesol by filtration

The binding of farnesol to seven filter types (all 25 mm in diameter) was measured by filtering 30 mL (3 × 10 mL) of GPP media containing 2 × standard A. A prefilter aliquot was taken for baseline values. All filters were purchased from Thermo Fisher Inc. (Waltham, MA, USA). They were the following: glass fiber GF/A Whatman 1820–024; Nylon 09-719C; surfactant-free cellulose acetate 190–9920; cellulose acetate Corning 430,636: polycarbonate Nucleopore Whatman 111,106; cellulose nitrate Whatman 7195–004; and mixed cellulose esters Celltreat Scientific Products 229,751. Each filter type was tested on 3 or 4 occasions in triplicate. Percent reduction values are presented as the mean of at least three independent experiments ± standard deviation. Both syringe and vacuum filtration were employed. GC analyte measurements were done following extraction.

### Statistical analysis

Statistical analyses were performed using Microsoft Excel (Version 16.61, Microsoft Office, Las Vegas, NV, USA) and GraphPad Prism Software (Version 9.3.1, San Diego, CA, USA). All biological data are represented as mean ± SEM of at least 3 biological replicates unless otherwise stated. Differences between two groups were accessed by unpaired two-tailed Student’s *t*-test. Differences were considered significant at *p* < 0.05. In cross comparing analytes for instrument validation, percent relative standard deviation (RSD%) is often reported.

## Results

### Method design

The primary goal of this work was to develop a high-throughput, quantitative assay for farnesol and aromatic fusel oils (phenethyl alcohol, tyrosol and tryptophol) measured in whole cultures, as well as pellet and supernatant fractions. This assay can, for example, be used to assess the levels of farnesol produced on a per cell basis throughout a complete growth curve. Given the need for technical and biological replicates at each time point, using this assay throughout a complete growth curve requires several hundred farnesol measurements for each culture studied. Thus, we needed a simple, scalable assay superior to those we had employed originally (Hornby et al. 2001; Hornby and Nickerson 2004). Critically, our method (Fig. 1) avoids both rotary evaporation and filtration. We have also included modifications intended to minimize lab to lab variations and variations among individual researchers in the same lab. The method employs GC-FID, measures farnesol directly rather than a derivatized farnesol, and uses 1-tetradecanol as an internal standard. It extracts the molecules of interest (farnesol, phenethyl alcohol, tyrosol, and tryptophol) into ethyl acetate rather than hexane in order to quantify all four molecules in the same assay. Hexane is a slightly better solvent for the extraction of farnesol (Hornby and Nickerson 2004; Weber et al. 2008, 2010), but it does not extract the aromatic fusel alcohols as well. This latter feature is highly desirable because there are many reports in the literature that aromatic fusel alcohols also alter fungal cell morphogenesis (Chen and Fink 2006; Rodrigues and Černáková 2020). Also, on previous occasions when we measured the farnesol retained in the cell pellet (Hornby and Nickerson 2004; Navarathna et al. 2005), roughly half of the farnesol remained cell bound. Now, we wanted to assess the relative importance of cellular associated farnesol by getting reliable measurements for both. The ability to compare whole culture values versus the sum of the pellet and supernatant fractions (WPS relative error Eq. 1) provided that reliability.

**Fig. 2:**
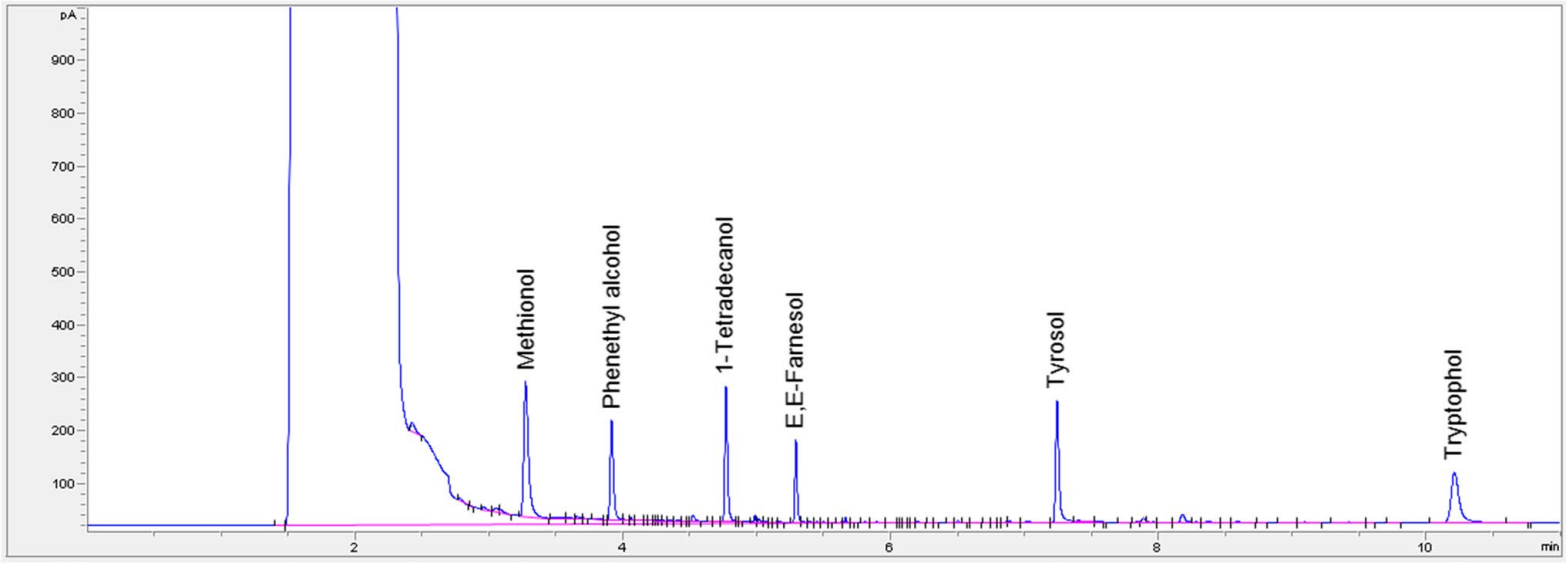
Representative GC chromatogram of standard A mix. The mix contains methionol (40 ng/μL), phenethyl alcohol (2 ng/μL), 1-tetradecanol (17.2 ng/μL), E,E-farnesol (1 ng/μL), tyrosol (15 ng/μL), and tryptophol (5 ng/μL)

To optimize analyte sensitivity, we compared sample volumes of 5 or 10 mL with extraction by 0.5–2.0 mL ethyl acetate, followed by 0.5–1.0 mL of 5 M NaCl or KCl, and various concentrations of 1-tetradecanol internal standard to give peak intensities close to those of the sample analytes, especially farnesol. Eliminating premeasurement steps now known to remove farnesol allowed us to use small sample and extraction volumes of 10 mL and 1.5 mL, respectively. Salt is added to decrease ethyl acetate aqueous solubility and improve the aqueous/ethyl acetate phase separation. The combination of a 10 mL sample with 1.5 mL of ethyl acetate and 1-tetradecanol as an internal standard at 17.2 ng/µL achieved the requisite cell lysis and phase separation, with appropriate GC signal intensities for biologically relevant concentrations of the analytes (Fig. 2). Microscopic examination following staining with trypan blue showed that > 99.9% of the yeast cells were killed by this treatment. This dual role for ethyl acetate is advantageous in that there is no time lag between cell lysis and extraction. To be sure that we had achieved complete cell lysis, we compared samples with and without a glass bead vortex step. Since no differences in trypan blue staining or analyte extraction were detected with and without vortexing, the glass bead step was omitted. The fusel alcohols derived from the branched-chain amino acids (isobutanol, isoamyl alcohol, and amyl alcohol) are more volatile than those derived from the aromatic amino acids and their GC peaks unfortunately are under the solvent peak for ethyl acetate.

### Physical loss of the farnesol via evaporation

We know that farnesol is volatile in that it is a common perfume ingredient (Nickerson et al. 2006), which becomes more evident during the culture of *C. albicans* mutants which overproduce farnesol (Kebaara et al. 2008). Additionally, farnesol was one of five volatile metabolites detected in the headspace above cultures of *C. albicans* and *C*. *dubliniensis* by headspace solid phase microextraction followed by GC/MS (Martins et al. 2007). We now found (Table 1) that half of the farnesol from uninoculated 250 mL flasks containing 50mL of standard growth media (GPP) spiked with 2 × standard A was lost by evaporation after 3 days of shaking at 225 rpm and 30 °C. This evaporative loss was dependent on the headspace/culture ratio. The loss was 56% in a flask which was open to the air but only 24% in flasks sealed with Duraseal® and Parafilm® (Table 1). For the three fusel alcohols, there were no significant decreases over the 72-h time frame (data not shown). Thus, our method carefully avoided concentration of the solvent extract by rotary evaporation.

**Table 1:** Percent reduction in farnesol following evaporation. Data are the means of % farnesol loss ± SD of two independent experiments

Time (hours)	Mean % loss farnesol ± SD	
	Unsealed	Sealed
24	26.41 ± 1.40	14.68 ± 0.66
48	43.53 ± 0.02	18.71 ± 0.45
72	56.40 ± 4.47	23.92 ± 2.74

### Physical loss of farnesol via filtration

Similarly, previous methods regularly included filtration to remove cells remaining after centrifugation without giving sufficient regard to the chemical composition of the filters and their ability to bind farnesol (Table 2). All seven of the filter types tested caused substantial reductions in the levels of farnesol detected in standard growth medium. The glass fiber filters gave the least reduction while the cellulose acetate filters gave the largest reduction (Table 2). Equivalent reductions were observed using vacuum filtration or syringe filtration (Table 2). This high level of removal by filtration was specific for farnesol in that none of the fusel alcohols tested showed greater than 5% reduction. Previous reports from this lab used cellulose nitrate filters (Hornby and Nickerson 2004; Navarathna et al. 2007). The experiments described in Table 2 were for a single concentration of farnesol (2 × standard A) in GPP growth medium. The % reduction would likely vary as the initial concentration of farnesol varied; we would expect a greater % reduction at the lower initial concentrations, reflecting a decreased binding capacity as the available surface area is covered in a monolayer (Couche et al. 1986).

**Table 2:** Percent reduction in farnesol following filtration^a^

Filter type	Mean % reduction ± SEM	N^b^
No filter	0 ± 1.82	3S
GF/A (glass fiber)	27.61 ± 0.59	3V
Nylon (0.22 µm)	55.86 ± 0.94	4S
Cellulose acetate (0.22 µm)^c^	80.47 ± 0.76	3S
Cellulose acetate (0.22 µm)	98.4 ± 0.20	3V
Polycarbonate nucleopore (0.22 µm)	48.13 ± 0.83	3V
Cellulose nitrate (0.22 µm)	71.06 ± 0.89	3V
Mixed cellulose ester (0.22 µm)	52.96 ± 1.74	3S

^a^From 10 mL samples of GPP media containing 2 × standard A

^b^Number of independent experiments. *S*, syringe filtered; *V*, vacuum filtered

^c^Filters were surfactant-free cellulose acetate

### Method validation

Five general experiments for validation were employed: Instrument precision and analyte specificity were conducted with the analytes (standard A) in water; linearity and range followed by assay robustness and reproducibility were conducted with the analytes (standard A and standard B) in water and two growth media (YPD and RPMI); and extraction efficiency and accuracy were conducted on whole cell cultures and fractionated parts spiked with known amounts of the analytes (standard A and standard B). Details are described in the next five sections.

### Instrument precision

A typical GC chromatogram showed well separated peaks for all analytes and the 1-tetradecanol internal standard (Fig. 2). Ten replicate injections of analyte and 1-tetradecanol were conducted with a sample of standard A. All RSD% values for mean area and ratio of analyte to 1-tetradecanol internal standard (IS) were under 3% with an average area RSD% for all analytes of 0.60 ± 0.84 SD, and average analyte to IS ratio of 1.55 ± 0.58 SD (Supplemental Table S1). The GC instrument is precise for analyte measurement.

### Analyte specificity

Chromatographic analyte separation was of little concern given the widely separate analyte retention times (Fig. 2). However, given the wide range of possible analyte concentrations, a series of 12 standard mixes containing biologically relevant concentrations were generated to detect any analyte signal interference. For each analyte the detection efficiency (analyte to IS ratio) was compared at both low and high concentrations, as defined by standard A and standard B, respectively (Supplemental Table S2-1). For instance, in the low comparison for phenethyl alcohol, the levels of phenethyl alcohol are determined when all 5 analytes are low (standard A) versus when only phenethyl alcohol is low, with the other four analytes at the standard B levels. Similarly, for the high comparison, the level of phenethyl alcohol when all 5 analytes are high (standard B) versus when only phenethyl alcohol is high, with the other four analytes at standard A levels. Each comparison, performed in triplicate, had %RSD for the analyte to 1-tetradecanol internal standard below 5% (Supplemental Table S2-2), suggesting that there is minimal to no signal interference within the biological range of these analytes, and that the assay is specific for analytes assessed.

### Linearity and range for biologically relevant concentrations of farnesol and aromatic fusel oils

**Fig. 3:**
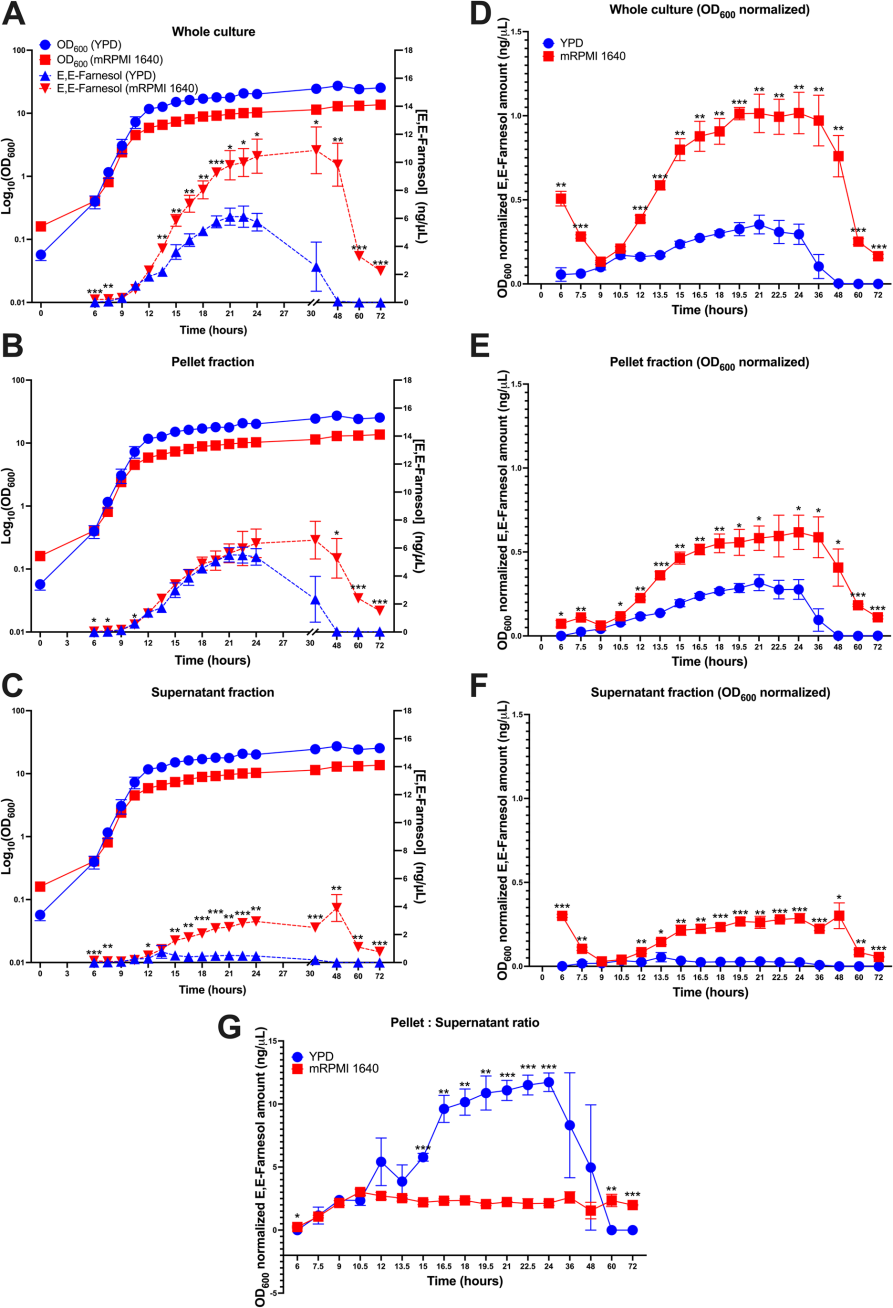
Production of farnesol in YPD and mRPMI 1640 over 72 h. Farnesol production was accessed in **A** whole culture, **B** pellet fraction, **C** supernatant fraction, **D** OD_600_ normalized whole culture, **E** OD_600_ normalized pellet fraction, **F** OD_600_ normalized supernatant fraction, **G** pellet to supernatant ratio. Data are the means ± SEM of three biological replicates. Note the change in the time scale before and after 24 h for **A**, **B**, and **C**. Student’s *t*-test was performed for comparison between YPD and mRPMI (*n* = 3, *P < 0.05, **P < 0.01, ***P < 0.001). YPD is displayed in blue and mRPMI is displayed in red, respectively

A useful assay should work best for biologically relevant analyte concentrations. To determine the appropriate range for the fusel alcohols, *C. albicans* was grown in minimal media containing the amino acid precursor for that alcohol with and without ammonium tartrate. To determine the range for farnesol, the ratio of the farnesol area to IS area for cultures grown in YPD and mRPMI was assessed after 12 and 24 h, identifying values around 1 ng/μL (4.49 μM) farnesol (Fig. 3).

To evaluate the linearity of each assay, standards A and B were diluted to make 8 concentrations of each analyte, exceeding the minimum suggestion of five by the International Council for Harmonization of Technical Requirements for Pharmaceutical for Human Use (ICH) (Bhardwaj et al. 2016). Given the wide variety of media used in *C. albicans* research, we determined the linearity and range for extraction from a complex medium (YPD), a defined medium (mRPMI), and water (Table 3). Eight concentrations of each analyte were extracted in triplicate from each. The correlation coefficients were calculated by plotting analyte to 1-tetradecanol ratios versus analyte concentrations and all were greater than 0.997. For illustration, the analyte plot and table for farnesol are displayed in Supplemental Fig. S1 and Supplemental Table S3. The detection limits (DL) were calculated using 3.3 * (SD/slope) and the quantification limits (QL) were calculated using 10 * (SD/slope). Table 3 shows these values for all five analytes from three growth media/solutions.

**Table 3:** Analyte observed and calculated linearity and dynamic range^a,b^

Analyte	Retention time	Detection limit (ng/µL)						Quantitation limit (ng/µL)						Upper limit (ng/µL)
		Observed			Calculated			Observed			Calculated			Observed
		Water	YPD	RPMI	Water	YPD	RPMI	Water	YPD	RPMI	Water	YPD	RPMI	
Methionol	3.30	6.25	0.80	1.00	4.05	2.91	2.41	12.50	3.87	0.40	12.27	8.82	7.31	150
Phenethyl alcohol	3.95	0.04	0.20	0.20	0.77	1.04	0.50	0.16	0.40	0.40	2.35	3.16	1.52	160
E,E-Farnesol	5.33	0	0.02	0.02	0.15	0.05	0.09	0.06	0.05	0.05	0.45	0.14	0.27	12
Tyrosol	7.31	0.04	0.90	0.90	2.30	1.48	1.51	0.16	4.48	0.45	6.98	4.48	4.58	45
Tryptophol	10.37	00.03	0.31	0.20	0.61	0.31	0.20	0.23	0.93	0.20	1.84	0.93	0.62	30

^a^Based on recovery of eight different concentrations of each analyte. As an example, data for farnesol are shown in Supplementary Table S3 and Supplementary Fig. S1. ^b^Dynamic range extends from quantitation limit to upper limit

These QL values are for the calculated quantification limits. However, there were also many acceptable (< 0.20) WPS relative errors for earlier time points in the growth curves prior to QL being attained (Supplemental Table S4). These observed QL values suggest a broader dynamic range than those calculated. The observed QL values were defined by extracting 1:10, 1:50, and 1:100 dilutions of standards A and B in triplicate from each growth medium (Table 3). Concentrations with a relative error at or below 0.10 linearly compared to their undiluted standard were considered to be in the dynamic range with an observed QL. These values were 0.4 ng/μL for phenethyl alcohol in both YPD and mRPMI, and 0.05 ng/μL for farnesol in both YPD and mRPMI (Table 3). The tryptophol and tyrosol calculated QL and observed QL were the same for YPD grown cultures, but quite different for the mRPMI grown cultures (Table 3). These differences show the importance of examining and comparing both complex and chemically defined growth media. Finally, the observed detection limits are based on signal intensities that are greater than twofold above the noise levels for that analyte (Table 3).

### Assay robustness and standard reproducibility

To assure assay robustness and reproducibility standards A and B were prepared fresh weekly. To confirm this reproducibility, the analyte to 1-tetradecanol ratios of six independent preparations from different working stocks were done by three different researchers. Standards extracted from both complex and defined media showed average analyte RSD% values as follows: farnesol 6.21, phenethyl alcohol 5.27, tyrosol 3.51, tryptophol 3.74, and methionol 3.75 (Table 4). Thus, the standard preparations are reproducibly quantitative for multiple users and are suitable for routine use.

**Table 4:** Assay robustness and standard reproducibility

Standard	Media	Prep	Methionol:IS ratio	Phenethyl alcohol:IS ratio	E,E-Farnesol:IS ratio	Tyrosol:IS ratio	Tryptophol:IS ratio
A	YPD	1	1.5134	0.8282	0.5085	1.1774	1.1662
		2	1.5521	0.8245	0.5300	1.2173	1.2925
		3	1.6070	0.8411	0.6115	1.2582	1.2170
		4	1.6216	0.9644	0.5970	1.2770	1.2208
		5	1.5991	0.8674	0.5886	1.2516	1.2715
		6	1.5395	0.7934	0.4913	1.2416	1.2340
		Average	1.5721	0.8532	0.5545	1.2372	1.2337
		SD	0.0431	0.0596	0.0508	0.0353	0.0445
		RSD%	2.74	6.98	9.17	2.85	3.61
	RPMI	1	1.6404	0.8626	0.4868	1.3081	1.2215
		2	1.7389	0.8710	0.5405	1.3115	1.2300
		3	1.6789	0.8637	0.6178	1.2103	1.1078
		4	1.7566	0.9971	0.5872	1.2865	1.0955
		5	1.6542	0.8686	0.5565	1.1833	1.0873
		6	1.6928	0.8454	0.5146	1.2334	1.1472
		Average	1.6936	0.8847	0.5506	1.2555	1.1482
		SD	0.0461	0.0558	0.0477	0.0541	0.0635
		RSD%	2.72	6.31	8.66	4.31	5.53
B	YPD	1	3.8134	15.1622	2.5819	3.5382	4.8995
		2	3.8553	15.2778	2.6492	3.6579	5.1300
		3	3.2805	14.5664	2.7555	3.5630	4.9772
		4	3.6047	15.2476	2.9447	3.8655	5.1300
		5	3.5648	16.2557	2.7498	3.6754	5.0096
		6	3.7362	14.8853	2.6034	3.7141	5.2483
		Average	3.6425	15.2325	2.7141	3.6690	5.0658
		SD	0.2106	0.5689	0.1342	0.1176	0.1267
		RSD%	5.78	3.73	4.95	3.21	2.50
	RPMI	1	4.1139	15.3830	2.7038	3.8495	5.0325
		2	4.0844	15.8329	2.6938	3.7712	5.0283
		3	3.7374	15.0023	2.6991	3.8130	4.7374
		4	4.0034	15.7146	2.8383	4.1199	4.7584
		5	3.8584	16.8205	2.7188	3.7290	4.6892
		6	4.0748	15.2868	2.6946	3.7732	4.9940
		Average	3.9787	15.6733	2.7247	3.8427	4.8733
		SD	0.1497	0.6367	0.0564	0.1419	0.1609
		RSD%	3.76	4.06	2.07	3.69	3.30
		Overall RSD%	3.75	5.27	6.21	3.51	3.74

### Fraction extraction efficiency and accuracy via % spike recovery

An assay’s accuracy and linearity can be determined by a spike recovery experiment wherein two cultures are compared, one of which has been spiked with a known standard while the other has not. How closely does the assay come to detecting the precise amount by which one exceeds the other? Also, given the vital importance of WPS relative error assessment in these assays, we wondered whether the % spike recoveries were equivalent for whole cultures, pellets, and supernatants. Thus, overnight YPD grown whole cultures and their pellet, and supernatant fractions were spiked in duplicate with both standard A and standard B and their individual recoveries were compared (Table 5). The overall percent analyte recoveries for both standards were: whole culture 97.08 ± 6.97, pellet 92.52 ± 8.02, and supernatant 102.81 ± 7.26 (Table 5). There was equivalent extraction and recovery for all three fractions and thus the whole cultures and fractions can be cross compared in analysis of their analyte measurement quality, i.e. their WPS relative error.

**Table 5:** % Spike recovery shows extraction efficiencies for whole cultures, pellets, and supernatants

Sample	Standard	Replicate	Methionol	Phenethyl alcohol	E,E-Farnesol	Tyrosol	Tryptophol
Whole culture	A	1	100.19	93.79	99.88	82.87	82.34
		2	101.95	97.43	109.87	88.35	89.34
	B	1	96.88	102.38	99.06	98.96	102.94
		2	98.65	93.88	97.89	100.01	104.92
		Average	99.42	96.87	101.68	92.55	94.89
		SD	2.16	4.05	5.52	8.33	10.86
Pellet fraction	A	1	86.17	91.01	103.07	76.96	85.14
		2	87.02	92.38	105.06	77.67	86.64
	B	1	89.61	91.49	99.41	95.08	102.62
		2	91.88	91.77	98.58	95.45	103.36
		Average	88.67	91.66	101.53	86.29	94.44
		SD	2.59	0.57	3.05	10.37	9.89
Supernatant fraction	A	1	109.01	100.75	99.14	95.93	89.73
		2	108.47	102.04	108.77	96.13	91.22
	B	1	112.84	112.43	100.00	115.19	109.71
		2	110.78	95.23	99.85	105.24	103.75
		Average	107.77	102.61	101.94	103.12	98.60
		SD	5.05	7.18	4.57	9.14	9.71

**Fig. 4:**
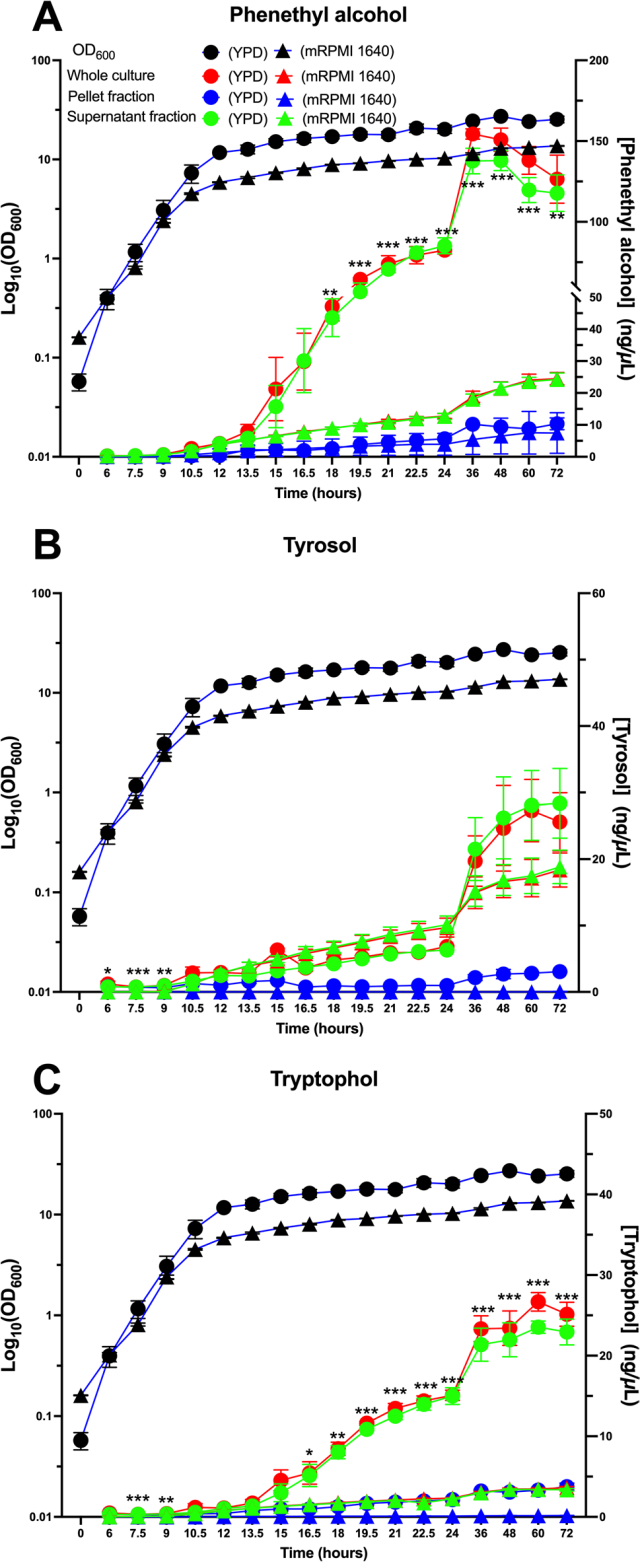
Production of aromatic fusel alcohol in YPD and mRPMI 1640 over 72 h. Phenethyl alcohol (**A**), tyrosol (**B**), and tryptophol (**C**) were accessed in YPD (circles) and mRPMI (inverted triangle) between the whole culture (red), pellet fraction (blue), and supernatant fraction (green). Data are the means ± SEM of three biological replicates. Student’s *t*-test was performed for comparison of whole culture fusel alcohol amounts between YPD and mRPMI (*n* = 3, *P < 0.05, **P < 0.01, ***P < 0.001)

The data in Table 5 were collected at a single time point. It is also important to check whether these accuracy and % spike recovery considerations apply to cells at all stages of growth. Thus, four samples were taken roughly 12 h apart throughout the growth of the YPD- and mRPMI-grown cultures (Figs. 3 and 4) and examined for their % spike recovery. Under all conditions farnesol extraction efficiency and percent recovery was calculated at 74% or greater (Supplemental Table S5). At high cell densities, such as in stationary phase, phenethyl alcohol was produced at very high amounts (≻ 150 ng/µL, Fig. 4A) and the assay was unable to differentiate the 2 ng/µL of standard A spike phenethyl alcohol from unspiked samples (Supplemental Table S5); however, the larger analyte concentration of standard B was recovered at 72% or greater. These experiments indicate that, as expected, assay sensitivity decreases as analyte concentrations reach significantly high levels. Overall, we demonstrate efficient and overlapping recovery in the complex matrix of cell growth across the dynamic range of the assay for all analytes.

### Method application

When considering the physiological significance of secreted molecules which are probably signaling molecules, it is important to quantify their concentrations at all stages of cell growth. Thus, we followed the production of farnesol and the aromatic fusel alcohols in triplicate for both YPD and mRPMI at 30 °C for 3 days (Figs. 3 and 4). Concentrations of the 4 analytes were measured in the whole culture, cell pellet, and supernatant at 90 min intervals over the growth phase (6 to 24 h) and at 12-h intervals thereafter. YPD and RPMI are two very different media commonly used for the growth of *C. albicans*. RPMI is a chemically defined medium containing all the amino acids, vitamins, and inorganic components typically needed by yeasts while YPD is a complex medium containing glucose, peptone, and yeast extract. The mRPMI replicates were supplemented with a mineral mix and buffered with 60 mM MOPS, pH 7.0. YPD was chosen to be part of this comparison because the chemical nature of its components, especially the peptone, would be focused at an ethyl acetate/aqueous interface, possibly interfering with analyte extraction and phase separation. However, the percent spike recoveries for the two growth media were comparable, with averages ± SD within 5% of each other (Supplemental Table S5). The secretion patterns varied greatly depending on the growth media (Figs. 3 and 4), as did the pellet:supernatant ratios (Fig. 3G) and the maximum analyte concentrations achieved (Table 6). These analyses, which are a direct consequence of the capabilities of our assay system, are the first to compare pellet and supernatant concentrations directly. They show that production and secretion are distinct and separable phenomena.

**Table 6:** Mean maximum analyte concentrations achieved in YPD and RPMI media over 72 h

Analyte	YPD	mRPMI
*E,E*-Farnesol	27.5 ± 2.7μM	46.6 ± 5.4 μM
Phenethyl alcohol	1263.9 ± 26 μM	220.7 ± 15.8 μM
Tyrosol	197.2 ± 30.2 μM	133.0 ± 18.5 μM
Tryptophol	165.4 ± 7.1 μM	22.52 ± 0.8 μM
Methionol	Below QL	Below QL

### Farnesol production over 72 h in YPD and mRPMI

The farnesol production data are shown in Fig. 3. The whole culture, pellet, and supernatant values are in Fig. 3A–C, respectively, and these values are normalized to cell growth (OD_600_) in Fig. 3D–F. The pellet:supernatant ratios over time are shown in Fig. 3G. As expected for a QSM, the whole culture farnesol values peaked in early stationary phase at approximately 6 ng/µL for growth in YPD and 10 ng/µL for growth in mRPMI. This difference between YPD and mRPMI grown cells was magnified when normalized on a per cell density basis (Fig. 3D) when the mRPMI grown cells produced 3 times more farnesol. Five other features of interest were also seen: (i) A majority of the farnesol remained in the cell pellet. The pellet to supernatant ratio for YPD grown cells reached almost 12 while the ratio for mRPMI grown cells was more constant at 4. (ii) The actual concentrations of farnesol in the cells must be much higher. For supernatants the farnesol should be equally distributed throughout 10 mL while for pellets the farnesol is constrained within the volume of the cells resuspended in 10 mL. (iii) Both culture types showed a rapid drop-off in farnesol levels later in stationary phase. This drop-off, which may be due partly to loss via evaporation (Table 1), was anticipated based on our prior observation of a similar decline in QSM activity in cell free supernatants during stationary phase (Hornby et al. 2001). (iv) The OD normalized values for farnesol in the cell pellets steadily increased ca. tenfold during cell growth (Fig. 3E), thus generating interest in the roles of intracellular farnesol in cell physiology and the mechanisms regulating farnesol secretion. (v) In contrast to the intracellular accumulation of farnesol, the OD normalized values for farnesol in the supernatants remained reasonably constant (Fig. 3F), as would be expected for a QSM whose concentration correlated directly with increasing cell mass. However, the farnesol per cell mass values for mRPMI grown cells were consistently 5–6 times greater than for YPD grown cells (Fig. 3F), in agreement with previous observations that extracellular farnesol levels varied greatly depending on the type of growth medium employed (Weber et al. 2010).

### Aromatic fusel alcohol production over 72 h in YPD and mRPMI

Figure 4 shows the aromatic fusel alcohol production data for whole culture, pellet, and supernatant fractions of both YPD and mRPMI grown cells. Data for phenethyl alcohol, tyrosol, and tryptophol are shown in Fig. 4A–C, respectively. Fusel alcohols are well known in yeasts as the byproducts of using amino acids as a less preferred nitrogen source, following removal of the nitrogen by a transaminase followed by the action of a decarboxylase, a dehydrogenase, and secretion (Hazelwood et al. 2008). Phenethyl alcohol, tyrosol, and tryptophol are made from Phe, Tyr, and Trp amino acids, respectively. Four points of interest were noted: (i) As expected, the fusel alcohols were largely secreted from cells; the whole culture and supernatant fractions closely paralleled each other for all three fusel alcohols (Fig. 4A–C). The concentrations present in the cell pellets were generally above the DL but below the QL for the first 15 h of growth. (ii) For all three fusel alcohols, synthesis started about 12 h into growth and continued for another 48 h. Thus, fusel alcohol synthesis coincided with stationary phase culture rather than rapid cell growth (Fig. 4). (iii) Phenethyl alcohol synthesis increased at a much higher rate (steeper slope) than did tyrosol or tryptophol, and the final levels of accumulation for phenethyl alcohol were 6 times higher (Fig. 3A). (iv) Whereas the mRPMI grown cells produced more farnesol (Table 6), it was the YPD grown cells which produced far more of the aromatic fusel alcohols (Table 6).

### Data measurement quality

The reliability of comparing whole culture values with the sum of the pellet and supernatant fractions (Eq. 1) is shown in Table 7. There are a total of 306 assay samples with values above their respective QL (Table 3) as follows: phenethyl alcohol 191 (62%), farnesol 233 (76%), tyrosol 121 (40%), and tryptophol 185 (60%) (Supplemental Table S4, green highlight). Methionol is left out of analysis because only 5 samples were above QL. Out of the 102 biological data points, all three assay samples were above their QL thresholds a total of: phenethyl alcohol 28 (27%), farnesol 72 (71%), tyrosol 0, and tryptophol 30 (29%). The average WPS relative error of each biological data point when all three assay samples were above the analyte QL are as follows: phenethyl alcohol 0.08 ± 0.05 SD, farnesol 0.11 ± 0.09 SD, tryptophol 0.08 ± 0.04 SD (Table 7). The standard deviation of the average relative error for farnesol is slightly skewed towards higher values given that 63 (88%) of the WPS data points within the dynamic range were less than 0.20, and 2 were above 0.30. We recommend that future applications of this method use a maximum WPS relative error value of 0.20. This cutoff point is especially important for single biological data points, such as a single extraction of a 24-h culture, while a higher cutoff point may be granted to time points that are supported by other data, such as those in a growth curve. All data points in the triplicate biological experiments for both YPD and mRPMI within the dynamic range are included here to display ruggedness (Table 7).

**Table 7:** Assay statistics; total assay samples 306, WPS biological data points 102. DR; dynamic range

Analyte	Assay samples in DR (*n* = 306)	WPS all in DR (*n* = 102)	WPS RE
Methionol	5 (2%)	0	NA
Phenethyl alcohol	191 (62%)	28 (27%)	0.08 ± 0.05
E,E-Farnesol	233 (76%)	72 (71%)	0.11 ± 0.09
Tyrosol	121 (40%)	0	NA
Tryptophol	185 (60%)	30 (29%)	0.08 ± 0.04

## Discussion

### The improved assay

The overall measurement quality of this assay is displayed in the WPS relative error values for each analyte (Eq. 1) at each biological data point. The method was fully validated in terms of precision, specificity, ruggedness, accuracy, solution stability, detection limits, quantitation limits, and overall system suitability. Key features include the following: (1) avoiding the filtration and evaporation steps which previously caused large losses of sample material. The amounts of farnesol removed following filtration (Table 2) and evaporation (Table 3) justify farnesol’s reputation as a “sticky” molecule and a perfume component and, in retrospect, make it surprising that the old assays were as informative as they were. (2) Use of 100% ethyl acetate for extraction which allows simultaneous cell lysis and measurement of all desired analytes. And (3) convenient comparison of a whole culture sample with a fractionated sample to measure both cell-associated (pellet) and cell-free (supernatant) values, while simultaneously justifying these values through their WPS relative error.

### Proof of principle — growth curves

Detailed time courses following farnesol and the fusel alcohols during batch culture have not been presented before. The experiments presented as proof of principle for the usefulness of this assay compared analyte production (total, pellet and supernatant) in two different growth media over a 72-h time course (Figs. 3 and 4). The assays are quantitatively reliable in that they identify and avoid traps which previously caused major underestimates of the farnesol present. For instance, previous reports (Nickerson et al. 2006) concluded that *C. albicans* only produced up to 2–4 µM farnesol in vitro whereas we now detect 17–27 µM (Table 3). These values are much closer to the higher values reported by Weber et al. (2008; 2010), even though they too filter sterilized their supernatants prior to assay. From these experiments, five general biological principles are apparent, two concern the fusel alcohols, and the remaining three concern farnesol: (1) the fusel alcohols are secreted immediately after they are synthesized. Note how the whole culture and supernatant values coincide (Fig. 4) while the pellet values are generally below the QL. (2) The three fusel alcohols are regulated in unison in that their syntheses start at 12 h (Fig. 4), occurring almost entirely during stationary phase rather than exponential phase. These features are consistent with their production via the Ehrlich pathway when the corresponding aromatic amino acids are used as less preferred nitrogen sources (Hazelwood et al. 2008; Ghosh et al. 2008). (3) In agreement with Weber et al. (2008, 2010), farnesol production varied greatly depending on the growth medium chosen; our mRPMI whole culture values exceeded YPD whole cultures by ca. twofold (Fig. 3A) and when normalized on a per OD basis the difference was ca. fourfold (Fig. 3D). (4) Farnesol synthesis and secretion are distinct and separable phenomena. Figure 3G shows that the pellet:supernatant ratio for YPD grown cells increases steadily to ca. 13 while that for the mRPMI stays roughly constant at 4. And (5) Farnesol levels begin to decline during stationary phase and diminish below DL (0.02 ng/µL) 24–48 h into stationary phase (Fig. 3). This drop in whole culture farnesol (Fig. 3A  and D) was accompanied or preceded by a dramatic loss of farnesol from the pellet fraction (Fig. 3E).

### Implications of the pellet: supernatant ratios for farnesol

During log growth, the per cell farnesol levels in the pellets increased ca. sixfold for mRPMI grown cells and threefold for YPD grown cells (Fig. 3E) whereas the OD normalized supernatant levels showed only a slight increase for the mRPMI cells and no increase for the YPD cells (Fig. 3F). When considering the physiological significance of a secreted molecule, it is important that a QSM should be secreted equivalently at all stages of cell growth so that the external concentration accurately reflects total cell number or cell mass (Hornby et al. 2001). The relatively constant values for the OD normalized supernatants (Fig. 3F) are consistent with farnesol’s function as a QSM. Similarly, the sharp increases in intracellular farnesol during growth (Fig. 3E) provide a reasonable explanation for a long-standing conundrum in fungal dimorphism known as the commitment phenomenon (Mitchell and Soll 1979; Chaffin and Wheeler 1981; Muthukumar and Nickerson 1985). Totipotent cells can differentiate as either yeasts or mycelia depending on the environmental conditions, often temperature, they are experiencing. Early in this differentiation process, if the cells are switched to the alternate environment, they will respond by growing in the alternate morphology. However, at a later point in time they are no longer able to switch; they have become committed to the morphology dictated by the original environment. The commitment phenomenon is likely shortened or non-existent for *eed1Δ* mutants studied by Polke et al. (2017). For wild type cells growing as yeasts at 30 °C, commitment may be determined by the increasing levels of intracellular farnesol.

### Loss of farnesol and/or QSM activity during stationary phase

A distinctive feature of the two time courses shown in Fig. 3A is the marked drop in farnesol during stationary phase. This decrease was expected because a similar drop in QSM activity had been observed in our original publication (Hornby et al. 2001). Note that Fig. 2 of Hornby et al. (2001) assessed QSM activity, i.e. the ability of culture supernatants to block mycelial development in a standard germ tube assay, rather than the presence of farnesol itself. This drop in farnesol and/or QSM activity (Hornby et al. 2001) could result from two scenarios. In the first, farnesol synthesis is turned off, since growth is no longer occurring, while farnesol secretion and evaporation continue. In the second, farnesol is converted, either spontaneously or enzymatically, into an inactive form. This transformation could be either intracellular or extracellular. Previously, we synthesized 40 analogs of farnesol (Shchepin et al. 2003). All these analogs had < 8% of farnesol’s QSM activity; very slight structural modifications rendered the analogs inactive. For instance, a possible oxidation product of farnesol, farnesol 10–11 epoxide, had only 1.7% of the activity of farnesol itself (Shchepin et al. 2003).

### Future applications

We anticipate that the availability of this reliable and accurate assay will permit a multitude of farnesol-directed questions to be addressed. A possible list includes the following: (1) how is farnesol synthesis regulated? High throughput screens of potential chemical inhibitors or transcription factor knock out mutant collections can be conducted. (2) All the experiments in this paper were conducted at 30 °C. Does farnesol production vary at 37 °C during germ tube formation and/or hyphal growth? (3) How does farnesol enter and leave the cell? Is this mechanism turned on in the *eed1Δ* mutant studied by Polke et al. (2017)? Does farnesol entry/secretion differ in the **a** and α haploid cells developed by Hickman et al. (2013)? Can this mechanism explain how *C. albicans* and *C*. *dubliniensis* evolved to secrete farnesol whereas other *Candida* species did not (Weber et al. 2008)? (4) We know that serum albumin binds farnesol as well as fatty acids, and that 50–100 × more farnesol is needed to block serum-induced GTF than GPP- or RPMI-induced GTF (Mosel et al. 2005). Is farnesol involved in the mechanisms by which serum triggers GTF and/or the mechanisms by which repeated cell washes reduce the concentration of serum required for GTF (Ahmad Hussin et al. 2016)? (5) What is the role of Zn(II) in cell growth, farnesol production, and the availability of totipotent cells for studying yeast/mycelia dimorphism (Mitchell and Soll 1979; Chaffin and Wheeler 1981). And (6) more speculatively, is farnesol involved in the white-opaque transition (Soll et al. 2003; Dumitru et al. 2007), or the conversion of commensal cells to pathogenic cells (Hube 2004)?

## Supplementary Information

Below is the link to the electronic supplementary material.

Supplementary file1 (PDF 302 KB)

## Data Availability

The datasets generated during and/or analyzed during the current study are available from the corresponding author on reasonable request. Growth curve raw data supplied in supplementary materials.
