# Supplementary material for: Quantitative assay for farnesol and the aromatic fusel alcohols from the fungus Candida albicans
Source: Appl Microbiol Biotechnol. 2022 Sep 15;106(19-20):6759–73. doi: 10.1007/s00253-022-12165-w (PMC9529689; doi:10.1007/s00253-022-12165-w)
Supplement: Supplementary file 1 — Supplementary file1 (PDF 302 KB) [file 253_2022_12165_MOESM1_ESM.pdf]

Applied Microbiology and Biotechnology

Quantitative assay for farnesol and the aromatic fusel alcohols from the fungus *Candida albicans*

Cory H.T. Boone*, Daniel J. Gutzmann, Jaxon J. Kramer, Audrey L. Atkin, and Kenneth W. Nickerson*

School of Biological Sciences
University of Nebraska
Lincoln, NE USA

*For correspondence. E-mail : knickerson1@unl.edu, boone.cory@huskers.unl.edu
Tel (402) 472-2253; Fax (402) 472-8722.

Supplementary Table S1. Instrument precision

Analyte	Mean area	Area RSD%	Mean analyte: IS area ratio	Ratio RSD%
Methionol	574.32	0.83	1.45	1.61
Phenethyl alcohol	297.12	0.34	0.75	0.76
1-Tetradecanol (IS)	395.32	0.92	-	-
E,E-Farnesol	193.59	1.53	0.49	1.94
Tyrosol	432.98	2.77	1.10	2.23
Tryptophol	430.30	1.13	1.09	1.20

Supplementary Table S2. Analyte specificity and standard composition

1 Standard concentrations			2 % Relative error analyte: IS ratio		
Analyte	[A] ng/µL	[B] ng/µL	Analyte	StdA: 1 Low(a)	StdB:1 High(b)
Methionol	40	100	Methionol	4.71	0.04
Phenethyl alcohol	2	40	Phenethyl alcohol	0.55	4.24
E,E-Farnesol	1	5	E,E-Farnesol	1.48	1.69
Tyrosol	15	45	Tyrosol	2.36	5.06
Tryptophol	5	20	Tryptophol	2.86	0.03

a/ comparison of indicated analyte detected in all low (standard A) versus 1 low where only that analyte is low, with the other 4 analytes at high standard B levels.

b/ comparison of the indicated analyte detected in all high (standard B) versus 1 high where only that analyte is high, with the other 4 analytes at low standard A concentrations.

Supplementary Table S3. Farnesol linearity and range determination; Supplementary Fig. S1. Farnesol linearity and range determination plot

	Water	YPD	RPMI
Conc. ng/ uL	E,E-Farnesol: IS Ratio		
0.05	0.02	0.03	0.03
0.1	0.05	0.05	0.05
0.5	0.24	0.25	0.25
1	0.50	0.51	0.50
2	1.02	1.04	1.04
3.5	1.71	1.73	1.72
5	2.43	2.50	2.52
7.5	3.90	3.90	3.83
	E,E-Farnesol: IS Ratio		
Ave. SD	0.02	0.01	0.01
LOD	0.15	0.05	0.09
LOQ	0.45	0.14	0.27

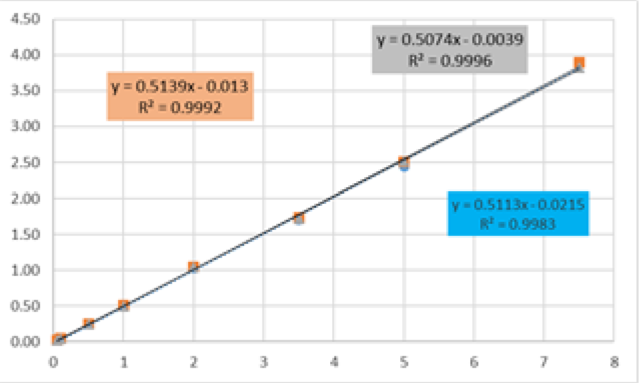

Supplementary Fig. S1.

Supplementary Table S4A. YPD raw GC values in ng μL^{-1} in YPD assay samples with WPS relative error

YPD		Phenethyl Alcohol			RE	E,E-Farnesol			RE	Tyrosol			RE	Tryptophol			RE
Bio Rep	Hours	W	P	S	W:(P+S)	W	P	S	W:(P+S)	W	P	S	W:(P+S)	W	P	S	W:(P+S)
	6	0.29	0.04	0.22	0.15	0.04	0.00	0.00	1.00	2.06	1.03	0.88	0.07	0.81	0.38	0.33	0.12
	7.5	0.35	0.01	0.33	0.04	0.06	0.04	0.03	0.17	0.67	0.66	0.75	1.10	0.32	0.28	0.30	0.83
	9	0.47	0.02	0.48	0.04	0.16	0.06	0.02	0.48	0.78	0.70	0.83	0.95	0.35	0.30	0.33	0.82
	10.5	3.66	0.39	1.26	0.54	1.11	0.36	0.19	0.51	4.57	1.37	1.39	0.40	2.08	0.49	0.50	0.53
	12	3.53	0.30	3.26	0.01	1.54	0.87	0.34	0.21	2.28	0.66	2.19	0.25	0.81	0.38	0.88	0.56
	13.5	5.02	0.63	4.90	0.10	1.74	1.46	0.28	0.01	2.35	1.24	2.40	0.55	1.15	0.47	1.16	0.41
	15	9.57	1.11	7.35	0.12	2.92	2.33	0.43	0.06	5.90	1.69	2.72	0.25	2.90	0.76	1.64	0.17
	16.5	15.28	0.96	15.72	0.09	3.85	3.24	0.39	0.06	2.86	0.74	3.11	0.35	3.16	0.63	2.99	0.15
A	18	38.42	1.76	34.04	0.07	5.26	4.63	0.51	0.02	3.94	0.90	3.92	0.22	6.86	1.01	6.57	0.11
	19.5	64.75	3.22	54.80	0.10	6.58	5.60	0.59	0.06	5.64	0.84	4.86	0.01	11.60	1.59	10.53	0.04
	21	73.13	4.32	69.09	0.00	6.81	6.21	0.59	0.00	5.84	0.79	5.86	0.14	13.30	1.74	12.90	0.10
	22.5	79.32	4.72	80.44	0.07	7.34	6.37	0.61	0.05	5.88	1.17	6.53	0.31	14.80	1.89	14.72	0.12
	24	82.87	5.23	87.97	0.12	6.71	6.45	0.61	0.05	7.19	1.18	6.41	0.06	15.92	2.04	16.82	0.18
	36	150.82	10.31	148.58	0.05	5.93	5.50	0.45	0.00	21.65	2.82	24.55	0.26	25.27	3.29	24.13	0.08
	48	151.88	9.60	147.80	0.04	0.16	0.11	0.01	0.25	35.11	3.80	35.29	0.11	25.58	3.05	24.81	0.09
	60	138.47	9.57	131.63	0.02	0.02	0.01	0.00	0.74	31.17	3.16	35.43	0.24	26.26	3.36	24.53	0.06
	72	138.19	8.31	132.01	0.02	0.01	0.05	0.00	7.50	30.38	3.07	35.03	0.25	26.43	3.17	25.35	0.08
	6	0.22	0.02	0.21	0.07	0.00	0.00	0.00	#DIV/0!	0.78	0.73	0.72	0.86	0.32	0.29	0.29	0.84
	7.5	0.27	0.02	0.25	0.01	0.04	0.00	0.00	1.00	0.73	0.68	0.70	0.89	0.31	0.27	0.29	0.82
	9	0.53	0.03	0.43	0.12	0.23	0.08	0.05	0.44	0.92	0.74	0.82	0.69	0.37	0.27	0.33	0.61
	10.5	1.55	0.19	1.36	0.00	0.92	0.43	0.21	0.31	1.61	1.05	1.36	0.51	0.59	0.32	0.49	0.37
	12	3.74	0.43	3.57	0.07	1.94	1.38	0.29	0.14	3.03	0.83	2.42	0.07	1.28	0.39	1.01	0.09
	13.5	6.46	0.78	6.19	0.08	1.94	1.70	0.33	0.05	2.83	1.28	2.48	0.33	1.42	0.56	1.39	0.37
	15	13.19	1.66	11.03	0.04	3.25	2.61	0.47	0.05	6.87	2.03	2.96	0.27	3.64	0.74	2.15	0.20
	16.5	28.22	1.46	25.27	0.05	4.18	3.44	0.39	0.08	3.42	0.70	3.32	0.17	5.23	0.86	4.71	0.07
B	18	49.21	2.54	42.25	0.09	4.70	4.01	0.44	0.05	4.06	1.11	3.83	0.22	9.06	1.32	8.11	0.04
	19.5	65.80	3.67	59.08	0.05	5.49	4.72	0.50	0.05	4.81	0.75	4.57	0.11	12.05	1.64	11.13	0.06
	21	83.22	5.05	74.64	0.04	6.65	5.80	0.57	0.04	5.54	0.82	5.36	0.12	14.61	1.93	12.95	0.02
	22.5	88.64	5.93	87.14	0.05	6.29	5.67	0.51	0.02	5.75	0.99	5.96	0.21	15.19	2.11	14.69	0.11
	24	86.30	6.58	91.96	0.14	5.92	5.24	0.46	0.04	6.67	0.90	6.31	0.08	15.48	2.28	14.91	0.11
	36	160.76	10.56	142.15	0.05	1.67	1.51	0.12	0.02	25.09	2.32	27.77	0.20	24.59	3.44	22.59	0.06
	48	162.72	9.68	139.43	0.08	0.08	0.01	0.00	0.85	25.86	2.86	28.82	0.23	25.52	3.26	23.24	0.04
	60	152.69	8.76	122.20	0.14	0.00	0.00	0.00	#DIV/0!	32.72	3.00	30.84	0.03	28.85	3.33	24.06	0.05
	72	144.22	8.69	125.79	0.07	0.00	0.03	0.00	#DIV/0!	29.54	3.24	32.07	0.20	26.82	3.48	23.55	0.01
	6	0.37	0.03	0.36	0.04	0.02	0.00	0.00	0.87	0.78	0.71	0.74	0.87	0.31	0.29	0.30	0.87
	7.5	0.51	0.04	0.51	0.07	0.12	0.05	0.02	0.43	0.89	0.74	0.80	0.74	0.34	0.27	0.32	0.77
	9	1.09	0.06	1.07	0.03	0.59	0.29	0.12	0.32	1.27	0.52	1.17	0.33	0.47	0.29	0.43	0.52
	10.5	2.55	0.20	2.45	0.04	1.60	1.06	0.34	0.13	2.45	1.41	2.09	0.43	0.85	0.36	0.68	0.23
	12	5.29	0.35	5.49	0.11	2.08	1.92	0.21	0.03	3.49	1.54	2.87	0.27	1.19	0.49	1.22	0.45
	13.5	12.29	5.14	6.42	0.06	2.91	2.04	1.67	0.27	3.11	2.21	2.37	0.47	2.60	1.33	1.50	0.09
	15	40.95	3.76	28.79	0.20	4.63	4.02	0.63	0.00	6.20	1.51	3.83	0.14	7.08	1.46	5.17	0.06
	16.5	45.99	3.54	49.39	0.15	5.32	5.01	0.43	0.02	4.49	0.78	4.72	0.23	7.97	1.50	7.71	0.15
C	18	53.71	3.54	54.47	0.08	5.35	5.01	0.41	0.01	6.45	0.78	4.97	0.11	9.44	1.50	9.40	0.15
	19.5	62.68	4.46	64.33	0.10	5.19	4.85	0.36	0.00	5.23	0.78	5.56	0.21	11.20	1.79	10.94	0.14
	21	65.25	4.34	68.26	0.11	4.91	4.52	0.36	0.01	6.32	1.07	5.88	0.10	12.47	1.81	11.62	0.08
	22.5	70.07	4.63	74.98	0.14	4.73	4.44	0.34	0.01	6.24	0.80	5.77	0.05	13.20	1.91	12.50	0.09
	24	78.07	5.20	75.62	0.04	4.55	4.41	0.34	0.04	6.66	0.81	6.20	0.05	13.84	2.10	13.23	0.11
	36	151.64	9.69	122.33	0.13	0.07	0.02	0.00	0.76	12.38	1.37	12.23	0.10	20.15	2.88	17.37	0.00
	48	137.92	8.85	126.49	0.02	0.01	0.00	0.00	1.00	12.92	1.44	14.48	0.23	19.14	2.90	17.86	0.08
	60	123.01	8.00	105.24	0.08	0.00	0.00	0.00	#DIV/0!	17.99	2.29	17.94	0.12	24.93	3.25	22.05	0.01
	72	96.88	14.04	95.42	0.13	0.00	0.02	0.00	#DIV/0!	16.89	2.86	18.06	0.24	22.17	4.60	19.91	0.11

Supplementary Table S4B. RPMI raw GC values in ng μL^{-1} in RPMI assay samples with WPS relative error

RPMI		Phenethyl Alcohol			RE	E,E-Farnesol			RE	Tyrosol			RE	Tryptophol			RE
Bio Rep	Hours	W	P	S	W:(P+S)	W	P	S	W:(P+S)	W	P	S	W:(P+S)	W	P	S	W:(P+S)
	6	0.28	0.00	0.24	0.16	0.19	0.04	0.13	0.09	0.00	0.00	0.00	#DIV/0!	0.03	0.00	0.02	0.10
	7.5	0.32	0.00	0.29	0.09	0.23	0.10	0.09	0.21	0.00	0.00	0.00	#DIV/0!	0.07	0.00	0.05	0.24
	9	0.56	0.00	0.52	0.07	0.33	0.15	0.08	0.30	0.09	0.00	0.01	0.90	0.12	0.00	0.11	0.09
	10.5	1.98	0.06	1.95	0.01	1.02	0.57	0.21	0.23	1.27	0.00	1.28	0.01	0.38	0.01	0.39	0.05
	12	3.89	0.13	3.80	0.01	2.48	1.41	0.58	0.20	2.99	0.00	2.91	0.03	0.76	0.03	0.70	0.03
	13.5	5.25	0.18	5.14	0.01	4.18	2.64	1.10	0.11	4.25	0.00	4.22	0.01	1.05	0.04	0.99	0.01
	15	6.64	0.23	6.62	0.03	6.54	3.83	1.87	0.13	5.24	0.00	5.30	0.01	1.28	0.05	1.26	0.03
	16.5	8.13	0.33	7.84	0.01	8.03	4.28	2.10	0.21	6.17	0.00	6.62	0.07	1.51	0.07	1.47	0.01
A	18	9.18	0.34	9.09	0.03	8.71	5.36	2.37	0.11	7.42	0.00	7.56	0.02	1.75	0.07	1.65	0.01
	19.5	10.46	0.43	10.22	0.02	9.76	6.02	2.67	0.11	8.33	0.00	8.72	0.05	1.88	0.09	1.81	0.01
	21	11.67	0.39	11.23	0.00	11.17	6.45	2.95	0.16	9.25	0.00	9.69	0.05	2.07	0.07	2.00	0.00
	22.5	12.72	0.55	12.36	0.02	11.49	7.71	2.94	0.07	10.21	0.00	10.57	0.04	2.18	0.11	2.13	0.03
	24	13.62	0.48	13.56	0.03	12.25	7.75	3.27	0.10	11.01	0.00	11.54	0.05	2.34	0.10	2.33	0.04
	36	20.14	0.69	19.28	0.01	12.11	7.63	2.68	0.15	16.66	0.00	16.85	0.01	3.11	0.13	2.98	0.00
	48	23.00	0.90	23.23	0.05	11.61	7.74	2.71	0.10	18.61	0.01	18.62	0.00	3.54	0.17	3.55	0.05
	60	25.31	0.83	24.70	0.01	3.72	2.55	1.28	0.03	19.33	0.00	19.26	0.00	3.74	0.14	3.41	0.05
	72	25.78	1.04	25.76	0.04	2.50	1.73	0.86	0.03	19.60	0.07	20.01	0.02	3.77	0.16	3.34	0.07
	6	0.33	0.00	0.29	0.12	0.22	0.03	0.12	0.29	0.00	0.00	0.00	#DIV/0!	0.06	0.00	0.03	0.43
	7.5	0.31	0.00	0.30	0.01	0.22	0.09	0.07	0.27	0.00	0.00	0.00	#DIV/0!	0.06	0.00	0.05	0.14
	9	0.58	0.00	0.58	0.00	0.30	0.15	0.06	0.30	0.22	0.00	0.11	0.49	0.14	0.01	0.05	0.57
	10.5	1.94	0.05	1.88	0.00	0.95	0.51	0.18	0.28	1.24	0.00	1.38	0.11	0.39	0.01	0.36	0.03
	12	3.82	0.13	3.88	0.05	2.03	1.19	0.49	0.17	3.27	0.00	3.37	0.03	0.71	0.03	0.71	0.05
	13.5	5.31	0.22	5.39	0.05	3.59	2.16	0.98	0.13	4.65	0.00	4.72	0.01	1.02	0.05	0.99	0.03
	15	6.88	0.24	6.67	0.00	5.92	3.34	1.67	0.15	5.35	0.00	5.59	0.04	1.28	0.06	1.21	0.01
	16.5	8.21	0.30	8.08	0.02	7.15	4.38	1.78	0.14	6.66	0.00	7.04	0.06	1.45	0.06	1.45	0.04
B	18	9.35	0.32	9.51	0.05	8.63	5.35	2.06	0.14	7.57	0.00	7.73	0.02	1.66	0.07	1.64	0.03
	19.5	10.66	0.45	10.83	0.06	9.32	5.53	2.62	0.13	8.74	0.00	8.59	0.02	1.82	0.09	1.84	0.06
	21	11.79	0.50	11.27	0.00	10.48	6.13	2.77	0.15	9.42	0.00	9.86	0.05	2.05	0.09	1.95	0.01
	22.5	12.55	0.57	12.73	0.06	10.35	6.59	2.80	0.09	10.41	0.00	10.69	0.03	2.13	0.11	2.10	0.04
	24	13.52	0.51	13.70	0.05	10.94	6.92	3.17	0.08	11.21	0.00	11.33	0.01	2.23	0.11	2.21	0.04
	36	20.98	0.75	19.98	0.01	12.90	8.18	2.55	0.17	17.96	0.00	18.08	0.01	3.19	0.14	2.99	0.02
	48	23.91	0.89	23.56	0.02	11.10	5.21	5.81	0.01	19.48	0.00	19.72	0.01	3.63	0.16	3.35	0.03
	60	26.41	0.85	25.06	0.02	3.06	2.39	0.72	0.02	20.54	0.00	20.97	0.02	3.30	0.15	3.36	0.06
	72	26.96	1.17	26.68	0.03	1.92	1.27	0.68	0.02	22.07	0.11	22.49	0.02	3.77	0.18	3.65	0.02
	6	0.32	0.00	0.23	0.28	0.21	0.01	0.12	0.37	0.00	0.00	0.00	#DIV/0!	0.07	0.00	0.03	0.61
	7.5	0.31	0.00	0.32	0.03	0.23	0.08	0.09	0.24	0.00	0.00	0.00	#DIV/0!	0.05	0.00	0.05	0.06
	9	0.53	0.00	0.58	0.09	0.32	0.14	0.08	0.32	0.00	0.00	0.00	#DIV/0!	0.15	0.00	0.14	0.04
	10.5	1.80	0.05	1.69	0.03	0.87	0.51	0.15	0.25	1.07	0.00	1.00	0.06	0.43	0.02	0.41	0.01
	12	3.65	0.11	3.66	0.03	2.31	1.38	0.42	0.22	2.21	0.00	2.21	0.00	0.89	0.04	0.87	0.01
	13.5	5.04	0.16	5.19	0.06	3.77	2.30	0.77	0.18	2.96	0.00	3.08	0.04	1.25	0.05	1.27	0.05
	15	6.24	0.24	6.18	0.03	5.10	3.03	1.18	0.17	3.42	0.00	3.89	0.14	1.49	0.07	1.45	0.02
	16.5	7.20	0.28	7.21	0.04	5.94	3.76	1.51	0.11	4.47	0.00	4.60	0.03	1.64	0.08	1.63	0.04
C	18	8.22	0.33	8.21	0.04	6.76	3.95	1.79	0.15	4.89	0.00	5.06	0.04	1.88	0.10	1.74	0.02
	19.5	9.15	0.32	9.06	0.02	8.74	3.74	2.05	0.34	5.26	0.00	5.66	0.08	2.09	0.08	2.02	0.01
	21	10.24	0.36	9.90	0.00	7.74	4.30	1.87	0.20	6.34	0.00	6.45	0.02	2.18	0.10	2.14	0.03
	22.5	10.95	0.36	10.65	0.00	8.10	3.61	2.70	0.22	6.70	0.00	6.90	0.03	2.41	0.09	1.00	0.55
	24	11.25	0.40	10.73	0.01	8.13	4.35	2.37	0.17	6.85	0.00	7.55	0.10	2.43	0.11	2.23	0.04
	36	15.31	0.56	14.83	0.00	7.56	3.89	2.27	0.19	10.23	0.00	10.66	0.04	2.87	0.13	2.96	0.08
	48	17.42	0.65	17.68	0.05	6.79	2.84	3.18	0.11	11.81	0.00	12.26	0.04	3.17	0.14	3.27	0.08
	60	20.40	0.78	20.91	0.06	3.14	2.27	1.31	0.14	11.58	0.00	12.25	0.06	3.33	0.16	3.39	0.07
	72	20.65	0.88	20.20	0.02	2.34	1.56	0.75	0.01	13.44	0.00	14.00	0.04	3.35	0.17	3.10	0.03

Supplementary Table S5. % Spike recovery for standards A and B

Standard	Media	Preparation	Methionol	Phenethyl alcohol	EE-Farnesol	Tyrosol	Tryptophol
A	YPD	1	97.31	93.23	88.34	77.37	100.31
		2	96.43	49.16*	88.48	78.47	94.65
		3	95.19	-199.14	75.05	83.42	91.89
		4	93.52	229.89*	102.90	77.53	106.27
		Average	95.62		88.69	79.20	98.28
		SD	1.64		11.37	2.85	6.37
	mRPMI	1	87.52	80.90	95.20	81.42	90.92
		2	90.13	83.46	106.94	79.81	92.43
		3	88.28	89.05	104.47	88.45	95.99
		4	90.89	90.23	107.68	91.47	100.88
		Average	89.21	85.91	103.57	85.29	95.06
		SD	1.57	4.46	5.75	5.57	4.43
B	YPD	1	74.12	81.33	83.28	78.69	86.41
		2	78.98	83.79	85.13	85.20	93.11
		3	71.13	72.94	73.83	74.87	82.45
		4	75.40	74.27	82.04	84.45	93.02
		Average	74.91	78.08	81.07	80.80	88.75
		SD	3.25	5.29	4.99	4.91	5.24
	mRPMI	1	91.88	89.08	92.15	94.56	98.72
		2	91.40	89.48	95.66	95.61	101.18
		3	94.09	86.96	96.37	95.81	101.20
		4	92.67	88.70	88.70	94.30	99.40
		Average	92.51	88.55	93.22	95.07	100.12
		SD	1.17	1.11	3.53	0.75	1.26
	Overall	Average	88.06	84.88*	91.64	85.09	95.55
		SD	8.39	6.19*	10.52	7.31	6.15

*indicates phenethyl alcohol samples unable to differentiate 2 ng/µL spike of standard A to unspiked sample. Those values not included in overall recovery averages.
